# Resistance Patterns among Multidrug-Resistant Tuberculosis Patients in Greater Metropolitan Mumbai: Trends over Time

**DOI:** 10.1371/journal.pone.0116798

**Published:** 2015-01-21

**Authors:** Alpa Dalal, Akshay Pawaskar, Mrinalini Das, Ranjan Desai, Pralhad Prabhudesai, Prashant Chhajed, Sujeet Rajan, Deepesh Reddy, Sajit Babu, T. K. Jayalakshmi, Peter Saranchuk, Camilla Rodrigues, Petros Isaakidis

**Affiliations:** 1 Jupiter Hospital, Thane, India; 2 Vasant Vihar, Thane, India; 3 Médecins Sans Frontières (MSF) / Doctors Without Borders, Mumbai, India; 4 GTB Hospital, Sewri, Mumbai, India; 5 Lilavati Hospital, Mumbai, India; 6 Fortis Hospital, Mumbai, India; 7 Bhatia Hospital, Mumbai, India; 8 Observer Research Foundation, Mumbai, India; 9 L. H. Hiranandani Hospital, Mumbai, India; 10 Dr. D. Y. Patil Hospital, Navi Mumbai, India; 11 P.D. Hinduja National Hospital and Medical Research Centre, Mumbai, India; Institute of Pathogen Biology, CHINA

## Abstract

**Background:**

While the high burden of multidrug-resistant tuberculosis (MDR-TB) itself is a matter of great concern, the emergence and rise of advanced forms of drug-resistance such as extensively drug-resistant TB (XDR-TB) and extremely drug-resistant TB (XXDR-TB) is more troubling. The aim of this study was to investigate the trends over time of patterns of drug resistance in a sample of MDR-TB patients in greater metropolitan Mumbai, India.

**Methods:**

This was a retrospective, observational study of drug susceptibility testing (DST) results among MDR-TB patients from eight health care facilities in greater Mumbai between 2005 and 2013. We classified resistance patterns into four categories: MDR-TB, pre-XDR-TB, XDR-TB and XXDR-TB.

**Results:**

A total of 340 MDR-TB patients were included in the study. Pre-XDR-TB was the most common form of drug-resistant TB observed overall in this Mumbai population at 56.8% compared to 29.4% for MDR-TB. The proportion of patients with MDR-TB was 39.4% in the period 2005–2007 and 27.8% in 2011–2013, while the proportion of those with XDR-TB and XXDR-TB was changed from 6.1% and 0% respectively to 10.6% and 5.6% during the same time period. During the same periods, the proportions of patients with ofloxacin, moxifloxacin and ethionamide resistance significantly increased from 57.6% to 75.3%, from 60.0% to 69.5% and from 24.2% to 52.5% respectively (p<0.05).

**Discussion:**

The observed trends in TB drug-resistance patterns in Mumbai highlight the need for individualized drug regimens, designed on the basis of DST results involving first- and second-line anti-TB drugs and treatment history of the patient. A drug-resistant TB case-finding strategy based on molecular techniques that identify only rifampicin resistance will lead to initiation of suboptimal treatment regimens for a significant number of patients, which may in turn contribute to amplification of resistance and transmission of strains with increasingly advanced resistance within the community.

## Introduction

India has one of the highest burdens of multidrug-resistant tuberculosis (MDR-TB) globally, with an estimated 64,000 patients having MDR strains among notified pulmonary TB cases in 2013, which would account for more than 20 percent of the estimated global burden of drug-resistant tuberculosis [1]. The increase in MDR-TB cases within the country has been acknowledged by the Revised National Tuberculosis Control Program (RNTCP) [2] and a multi-faceted response plan has been prepared to combat this challenge [3].

A number of studies have described the prevalence of MDR-TB in a number of different geographical locations across the country [4–11]. The overall magnitude of the MDR-TB epidemic in Mumbai, one of the most known Indian mega-cities, is increasingly being documented. D’Souza et al in 2009 reported epidemic levels of drug resistance amongst new TB cases in central Mumbai [5]. In 2011 a cases series of totally-drug resistant TB (TDR-TB) in the city captured the attention of the medical community and the international media [12,13]. In late 2014, a survey among HIV-infected patients in the greater metropolitan Mumbai reported both alarming levels of drug-resistant tuberculosis among new and previously treated TB cases, as well as complex resistance patterns, especially high levels of resistance to fluoroquinolones and other second line anti-TB drugs [14]. However, as past studies have tended to emphasize the diagnosis, treatment and care of MDR-TB, there is a dearth of literature highlighting variations in resistance patterns over the years among selected populations.

The aim of this study was to document the patterns of drug resistance observed in MDR-TB patients receiving anti-tuberculosis treatment in Mumbai, India and to identify the trends in these patterns over time.

## Methods

### Ethics

The study was approved by the Institutional Ethics Committee of Jupiter Hospital in Mumbai, India. As this was a retrospective analysis of routinely collected data, written informed consent was not obtained from the study participants. The named ethics committee specifically approved the study and waived the need for consent. Patient information was anonymized and de-identified prior to analysis. The study also satisfied the criteria for reports using routinely collected programmatic data set by the Médecins Sans Frontières (MSF) Ethics Review Board, Geneva, Switzerland.

### Study design

The study was a retrospective, observational study using routinely collected laboratory data.

### Setting and study population

The study involved pooling data from eight medical facilities in Mumbai and the greater metropolitan area: Jupiter Hospital, Bhatia Hospital, Dr. D. Y. Patil Hospital, Fortis Hospital, L. H. Hiranandani Hospital, and Lilavati Hospital are multi-facility private hospitals in Mumbai and surrounding areas, each having a specialty unit for pulmonary disorders and tuberculosis. The Médecins Sans Frontières (MSF) clinic is a charity-run facility located in the western suburbs of Mumbai, which has been providing care and treatment for drug-resistant TB and HIV free of charge since 2006. The Sewri TB hospital is a government hospital that provides treatment to the largest cohort of MDR-TB patients in Mumbai with hospital wards designed to accommodate tuberculosis patients.

All eight facilities included in the study were following standard international and national guidelines on diagnosis and treatment of MDR-TB throughout the study period.

All patients enrolled in this study were suffering from bacteriologically confirmed MDR-TB and were attending one of the eight study facilities between January 2005 and December 2013.

### Tuberculosis resistance profiles

Resistance patterns were identified using drug susceptibility testing (DST). Only those patients whose specimens where tested for both 1^st^ and 2^nd^ line DST at an accredited lab were considered for inclusion in the study. In the earlier years the 2^nd^ line DST included only ofloxacin and kanamycin; newer fluoroquinolones and injectables were added in the last years. Hinduja Hospital laboratory was selected as the source of results as it is the only laboratory accredited by the Revised National Tuberculosis Control Programme (RNTCP) in Mumbai (including accreditation for 2^nd^ line DST since December 2013); having a universal source of DST results reduced discrepancies in data due to multiple standards. All DST were carried out using the MGIT 960 TB system.

A case of multidrug-resistant tuberculosis (MDR-TB) is defined as sputum/isolate in which the culture was positive for *Mycobacterium tuberculosis* and found to have *in vitro* resistance to isoniazid and rifampicin with or without resistance to other anti-tubercular drugs based on additional DST results.

Pre-extensively drug-resistant tuberculosis (pre-XDR-TB) patient is defined as an MDR-TB case whose recovered *M.* *tuberculosis* isolate is resistant to at least isoniazid, rifampicin, plus either a fluoroquinolone (ofloxacin, levofloxacin, or moxifloxacin) or a second-line injectable anti-TB drug (kanamycin, amikacin, or capreomycin).

Extensively drug-resistant tuberculosis (XDR-TB) patient is defined as having an MDR-TB strain that is resistant to at least isoniazid, rifampicin, as well as any fluoroquinolone (ofloxacin, levofloxacin, or moxifloxacin) and at least one second-line injectable anti-TB drug (kanamycin, amikacin, or capreomycin).

Extremely drug-resistant tuberculosis (XXDR-TB) patient is defined as a case of XDR-TB with additional resistance to any group V anti-TB drugs (e.g. para-aminosalicylic acid, ethionamide, clofazimine, linezolid).

### Management of patients diagnosed with drug-resistant TB

All patients diagnosed with MDR-TB and XDR-TB were managed in accordance with the national treatment guidelines [2], while those with pre-XDR TB and XXDR-TB (currently not included in the national guidelines) were offered individualized treatment regimens consisting of four anti-TB drugs likely to be effective, whenever possible.

### Data collection and statistical analysis

Clinical and laboratory data from the different facilities were pooled and merged into a single database, which was analyzed. To improve interpretability, we collapsed the data across 3 year-periods (2005–2007, 2008–2010, 2011–2013) to identify any periodic trends in the resistance patterns. We also used the moving averages method to increase stability and smooth the data.

We used the Mantel-Haenszel linear-by-linear association chi-square test and Poisson regression models to assess whether the changes in proportions of the different resistance patterns over time were statistically significant. A p value of <0.05 was considered significant. Data analysis was conducted with SPSS (IBM Corp. Released 2012. IBM SPSS Statistics for Windows, Version 21.0. Armonk, NY: IBM Corp.)

## Results

### Patient characteristics

Reports from a total of 340 MDR-TB patients tested between January 2005 and December 2013 were included in this study. The age distribution of patients was as follows: 0–15 years 33 (9.7%), 16–25 years 109 (32.1%), 26–35 years 94 (27.6%), 36 years and above 104 (30.6%). There were 188 males (55.3%), 150 females (44.1%) and two male-to-female transgender patients (0.6%).

### Drug resistance patterns

Throughout the nine years of the study period, the proportion of study participants having MDR-TB strains was 29.4%. Pre-XDR-TB was the most common form of drug-resistant TB in this Mumbai population at approximately 57% of the entire cohort. The proportions of XDR- and XXDR-TB were 9.7% and 4.1% respectively (Table 1).

**Table 1:** Resistance patterns among MDR-TB patients diagnosed among 8 facilities in greater metropolitan Mumbai, 2005–2013.

Year	2005–2007	2008–2010	2011–2013	Total	% Proportion among total sample
**MDR**	13	32	55	100	29.4%
Pre-XDR	18	64	111	193	56.8%
XDR	2	10	21	33	9.7%
XXDR	—	3	11	14	4.1%
Total	33	109	198	340	100%

Table 2 details the resistance patterns as recorded for each first and second line anti-TB drug over time. Of note was that 235/340 (69.1%) and 179/287(62.45) of patients tested for Ofloxacin and Moxifloxacin respectively were found to be resistant. Table 3 provides data on the additional resistance recorded among the MDR-TB patients; 35 (10.3%) of patients had developed resistance to 8 or more drugs, beyond the rifampicin and isoniazid while 55 (16.2%) patients were resistant to all fluoroquinolones and injectable drugs.

**Table 2:** Resistance profile (first and second-line anti-TB drugs) for MDR-TB patients in greater metropolitan Mumbai, 2005–2013.

Resistance profile	MDR-TB patients			
	2005–2007	2008–2010	2011–2013	Total
	n (%)	n (%)	n (%)	n (%)
**MDR-TB patients (N)**	**33**	**109**	**198**	**340**
**Any resistance to first-line drugs**				
S	27 (81.8)	96 (88.1)	179 (90.4)	302 (88.8)
H	32 (97.1)	108 (99.1)	197 (99.5)	337 (99.1)
R	33 (100)	109 (100)	198 (100)	340 (100)
E (N = 339)	22 (66.7)	78 (72.2)	156 (78.8)	256 (75.5)
Z (N = 309)	10 (43.5)	71 (76.3)	158 (81.9)	239 (77.3)
**Any resistance to second-line drugs**				
Ofx	19 (57.6)	67 (61.5)	149 (75.3)	235 (69.1)*
Mfx (N = 287)	6 (60.0)	41 (47.1)	132 (69.5)	179 (62.4)*
Am (N = 292)	-	12 (13.0)	29 (15.3)	41 (14.0)
Cm	-	10 (11.5)	25 (13.2)	35 (12.4)
Km (N = 339)	4 (12.5)	17 (15.6)	35 (17.7)	56 (16.5)
Eto	8 (24.2)	60 (55.0)	104 (52.5)	172 (50.6)*
Cfz (N = 282)	-	2 (2.4)	3 (1.6)	5 (1.8)
PAS (N = 339)	8 (25.0)	28 (25.7)	48 (24.2)	84 (24.8)

S-streptomycin, H-isoniazid, R-rifampicin, E-ethambutol, Z- Pyrazinamide, Ofx-ofloxacin, Mfx-Moxifloxacin, Km-kanamycin, Am-Amikacin, Cm-capreomycin, Eto-ethionamide, Cfz- Clofazimine, PAS- para-aminosalicylic acid

*Linear-by-linear association chi-square test, p value<0.05.

**Table 3:** Additional resistance to first and second-line anti-TB drugs among MDR-TB patients in greater metropolitan Mumbai, 2005–2013.

Resistance profile	MDR-TB patients (N = 340) n (%)
**Number of additional anti-TB drugs to which MDR-TB patients were resistant**	
0^*^	8 (2.4)
1	22 (6.5)
2	35 (10.3)
3	50 (14.7)
4	59 (17.4)
5	62 (18.2)
6	42 (12.4)
7	27 (7.9)
8 and above	35 (10.3)
**Additional second line anti-TB drugs to which MDR-TB patients were resistant**	
Ofx	89 (26.2)
Km	3 (0.9)
Eto	25 (7.6)
Ofx+Eto	99 (29.1)
Km+Eto	5 (1.5)
Ofx/Mfx+Km/Am/Cm	55 (16.2)

* Resistance to rifampicin and isoniazid only, Ofx-ofloxacin, Mfx-Moxifloxacin, Km-kanamycin, Am-Amikacin, Cm-capreomycin, Eto-ethionamide.

### Trends over time

**Figure 1:**
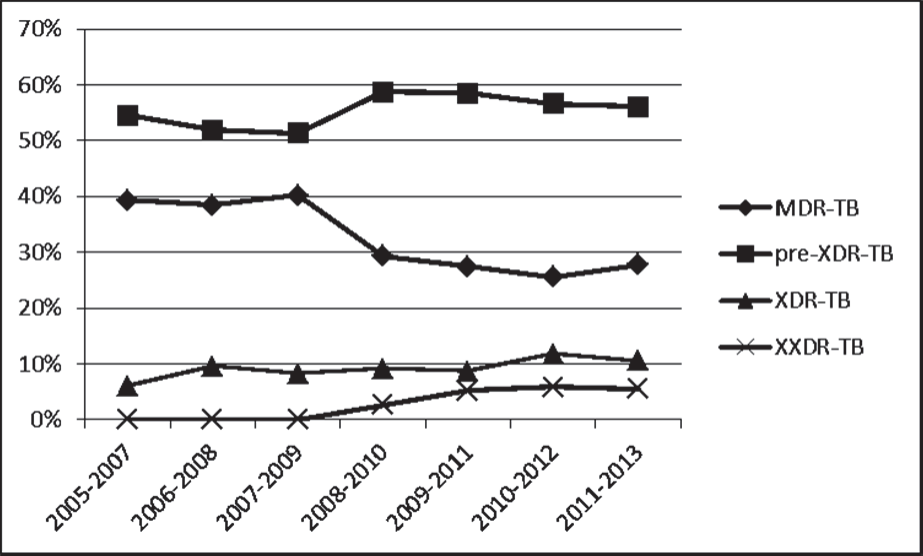
Trends over time of resistance patterns among DR-TB patients diagnosed in 8 facilities in greater metropolitan Mumbai, 2005–2013

The trends of the different forms of MDR-TB over time were not found to be statistically significant using a Mantel-Haenszel linear-by-linear association chi-square test (for preXDR; x^2^ = 0.012, p = 0.91, for XXDR; x^2^ = 2.96, p = 0.08) and Poisson regression models. It was observed that the proportion of patients with MDR-TB decreased from 39.4% in the period 2005–2007 to 27.8% in 2011–2013, while the proportion of patients with XDR-TB increased from 6% in 2005–2007 to 10.6% in 2011–2013 and the proportion of patients with XXDR-TB increased from 0% in 2005–2007 to 5.5% in 2011–2013 (Fig. 1).

Moreover, the proportions of patients with additional resistance to ofloxacin, moxifloxacin and ethionamide significantly increased over time. The linear-by-linear association chi-square-values and p-values were x^2^ = 7.87, p = 0.005 for ofloxacin, x^2^ = 9.19, p = 0.002 for moxifloxacin and x^2^ = 4.14, p = 0.04 for ethionamide (Table 2).

## Discussion

To our knowledge, this is the first study of multidrug-resistant tuberculosis (MDR-TB) trends over an extended period of time (2005–2013) in greater metropolitan Mumbai.

The six-fold increase in the absolute number of MDR-TB cases (Table 1) identified among the eight study facilities between the first 3-year period (2005–2007) and the last 3-year period (2011–2013), as well as the trend towards increasingly advanced patterns of resistance (i.e. XDR- and XXDR-TB), were both most likely due in large part to improving access to 1^st^ and 2^nd^-line DST. Other possible reasons include increasing awareness amongst clinicians of the possibility of MDR-TB and the need to send specimens for DST. Regardless, these data showing increasingly advanced resistance patterns represents a ‘wake up call’ to all of us working to combat TB in Mumbai and in other parts of the country.

Just as concerning is the finding that the most common form of drug-resistant TB in this study population was not MDR-TB but pre-XDR-TB, which is mainly due to the high levels of baseline fluoroquinolone resistance that are present in Mumbai [14,15]. This predominance of pre-XDR-TB has a number of important implications: firstly, the current treatment strategy that relies on standardized regimens to treat a traditional classification of MDR-TB carries a high risk of being suboptimal treatment. In many cases, a standardized MDR-TB regimen will not contain ‘four drugs likely to be effective’, as recommended by the WHO, if prescribed to a person with disease due to a pre-XDR-TB strain [16]. Not only does a suboptimal treatment regimen reduce the likelihood of a cure, but it can also lead to amplification of drug resistance, such that the pre-XDR-TB strain becomes an XDR-TB strain, which in turn can be transmitted within the household and community.

Secondly, all patients at risk of suffering from active TB disease due to a drug-resistant strain need to have drug susceptibility testing (DST) performed not just against first-line anti-TB drugs, but also against flouroquinolones and other second-line anti-TB drugs during the initial work-up. The current trend in India is to move towards routine use of a molecular TB diagnostic, for example Xpert MTB/RIF (also known as GeneXpert), which can rapidly detect rifampicin resistant strains of Mycobacterium tuberculosis within 2 hours. Although this can be viewed as an important step forward, it should be acknowledged that use of GeneXpert in settings like Mumbai may contribute to suboptimal prescribing practices, since detection of rifampicin resistance alone may mask a diagnosis of pre-XDR or XDR-TB (or worse). In settings like Mumbai, GeneXpert alone will not control the MDR-TB epidemic: TB culture and full DST must accompany its implementation. This will require an increasing state and government investment in laboratory capacity in order to meet the needs of TB patients and clinicians.

Thirdly, inappropriate prescribing patterns related to active TB disease in the private health sector and easy/over-the-counter access to fluoroquinolones both need to be halted as soon as possible. Although high levels of amplification of drug resistance have been demonstrated under well-established DOTS programme conditions [17], additional factors are contributing to the MDR-TB situation in Mumbai, namely a chaotic and unregulated private health sector that operates in parallel with the public DOTS programme, in which suboptimal TB treatment regimens are commonly being prescribed [18–20].

The results we have observed are in a sample population spread out over the cities of Mumbai, Navi Mumbai and Thane. Given the high population density in these settings, where a large proportion of the population lives in extreme poverty in slums, these data are unlikely to be representative of the state of Maharashtra and even less likely of the whole of India. However, the living conditions in Mumbai and the common practices in the private health sector (i.e. the prescribing of inappropriate TB treatment regimens and over-the-counter availability of fluoroquinolones and other drugs having anti-TB properties) are similar to those of other large metropolitan centres in the country.

This study is subject to the usual limitations inherent with retrospective design and data collection. Our sample size is small and may be not fully representative of the population of DR-TB patients within the setting. The extremely limited laboratory capacity in Mumbai prevented us from having a larger sample; until 2011 there was only one accredited laboratory in Mumbai for DST to first-line anti-TB drugs (which was private) and until December 2013 there was no accredited laboratory for DST to second-line drugs. Moreover we believe that there were several cases of MDR-TB cases that did not undergo 2^nd^ line DST, as the cost had to be covered by the patients themselves, and therefore the representativeness of our study may be further limited. We feel that the numbers of cultures and tests performed during the study period were abysmal, considering the size of the population and the magnitude of the MDR-TB epidemic in Mumbai. As the study was limited to bacteriologically confirmed patients, specific populations, especially small children and HIV-infected patients with culture negative tuberculosis might have been underrepresented in this study. The lack of statistical significance in the trends analysis can be attributed to the small sample size; we believe that the descriptive statistics illustrate the trends over time in drug resistance patterns but they should be interpreted with caution. We were not able to analyze patient characteristics even though we had access to individual patient data rather than aggregated data. However, we had access to limited demographic and clinical data of individual patients. We were also not able to ascertain whether some patients were tested more than once during the study period. However, considering the small size of the cohort in each facility, individual patients were easily identifiable by the treating physicians who kept their records up-to-date and therefore double entries were rather uncommon. Last, we had no access to fingerprinting data for this population, which could have complemented our description of the circulating strains. Despite these limitations we believe that our study adds important data and insights into the description of the drug-resistant tuberculosis epidemic in metropolitan Mumbai.

We emphasize the importance of developing a robust surveillance system for drug resistance in the city and the country rather than depending on infrequent national and limited sub-national surveys. National surveys albeit scientifically acceptable, they may mask significant variance in the magnitude of the epidemic in various localities areas or specific populations. This is especially relevant for vast countries like India and mega-cities like Mumbai where several drug-resistant TB epidemics may exist.

Ongoing research is necessary to provide an evidence base for appropriate public health action. Use of targeted molecular-guided cluster analyses should be considered in order to identify patterns of transmission at the population level. In the face of growing proportions of pre-XDR-TB, XDR- and XXDR-TB, these efforts must be initiated promptly. Official recognition and categorization of TB strains that are resistant to three or more anti-TB drugs (e.g. pre-XDR-TB) are needed, not only to standardize research efforts, but also to help ensure that these patients receive appropriate treatment regimens.

## Conclusion

The observed trends in TB drug resistance patterns in metropolitan Mumbai highlight the need for individualized drug regimens, designed on the basis of full drug susceptibility results to first- and second-line anti-TB drugs. A drug-resistant TB case-finding strategy, based solely on rapid molecular techniques such as GeneXpert that identify only TB with Rifampicin resistance, may contribute to suboptimal treatment regimens for the many patients suffering from disease due to pre-XDR-TB strains, and may lead to amplification resistance and spread of further worse forms of drug-resistant TB within the community.
